# SOP: First-ever epileptic seizure in adult patients

**DOI:** 10.1186/s42466-019-0006-4

**Published:** 2019-02-28

**Authors:** Julian Bösel

**Affiliations:** 1 Department of Neurology, Klinikum Kassel, Mönchebergstr. 41-43, 34125 Kassel, Germany; 2 Kassel School of Medicine, University of Southampton, Mönchebergstr. 41-43, 34125 Kassel, Germany

## Abstract

**Background:**

About 5% of all adults will have at least one epileptic seizure in their life. The incidence of all unprovoked seizures ranges from approximately 50 to 70 /100,000. The very first epileptic seizure in an adult can be a very decisive event and demands a great deal of responsibility on the part of the treating clinician. Optimal clinical work-up and systematic decision-making are necessary to ensure adequate treatment as well as to avoid unnecessary treatment, such as life-long application of anticonvulsants that may not be indicated.

**Aim:**

To present a pragmatic standard operating procedure (SOP) for approaching the first seizure in adults.

**Method:**

Based on current recommendations and personal suggestions, an SOP in the form of a flow chart accompanied with topical explanations and tables was created.

**Results:**

Approaching the first seizure should start with obtaining bystander information on the seizure and its clinical features. Then, differential diagnoses should be considered. The diagnostic work-up hast to contain a neurological and physical examination, emergency blood tests and cerebral imaging. This should allow to differentiate an unprovoked from an acute symptomatic seizure, i.e. triggered by current specific and identifiable structural or metabolic cause that should be eliminated if possible. In the case of an unprovoked seizure, estimation of seizure recurrence is necessary for the decision to start treatment with antiepileptic drugs.

**Conclusion:**

The challenge of diagnostic work-up and treatment decisions after a first epileptic seizure in adults may be facilitated by a systematic, SOP-based approach.

## Introduction

About 5% of all adults will have at least one epileptic seizure in their life. The incidence of all unprovoked seizures ranges from approximately 50 to 70 / 100,000 and that of epilepsy from 30 to 50 / 100,000 in the USA, UK or Europe [1]. The very first epileptic seizure in an adult can be a very decisive event and demands a great deal of responsibility on the part of the treating clinician. Statistically, it takes about four years in average until that person regains “normality” in both private and professional life, even if that first seizure is not followed by more. Optimal clinical work-up and systematic decision-making are necessary to ensure adequate treatment as well as to avoid unnecessary treatment, such as life-long application of anticonvulsants that may not be indicated. Because such work-up and decision-making is challenging and in several aspects controversial, dedicated first seizure clinics have been suggested and introduced in some countries [2]. The following standard operating procedure (SOP) will mainly cover the initial approach to an adult patient with a first seizure in the emergency room and the first days after admission, as based on current recommendations [3–5]. It is, however, not reflecting a particular guideline or approved by an expert committee, hence contains subjectivity to some degree. Further aspects such as details of drug choice, psychosocial adjunctive treatment, limitations of daily activities / profession / driving, etc. are beyond the scope of this article and will just be briefly addressed by reference to the current literature. Also, pediatric seizures are not discussed here. A very informative and practical approach along guiding questions can be found in this review: [6].

## Definitions

### Epilepsy

Chronic brain disease that is characterized by at least one of these conditions: 1. At least two unprovoked seizures or reflex-seizures with an interval of at least 24 h; 2. An unprovoked seizure with a recurrence risk of at least 60%; 3. The diagnosis of a specific epilepsy syndrome [3, 4].

### Epileptic seizure

Transient clinical signs or symptoms due to a pathologic excessive or (over-)synchronized neuronal activity in the brain [3].

### Acute symptomatic seizure

Seizures that result from some immediately recognizable stimulus or cause, i.e. that occur in the presence or close timely association (about a week) with an acute brain insult (metabolic, toxic, structural, infectious, hypoxic, etc.) [7].

### Unprovoked seizure

Seizures that do not require an immediate precipitating event, suggesting the possibility of an underlying neurological disorder and may particularly predispose to recurrent seizures [8].

## First things first: Immediate measures

- Check and secure vital functions
- Get a (medical) history from observer(s)
- Don’t use / prescribe anticonvulsants prematurely

## Flow chart SOP first seizure in adults

(Figure 1)

**Fig. 1:**
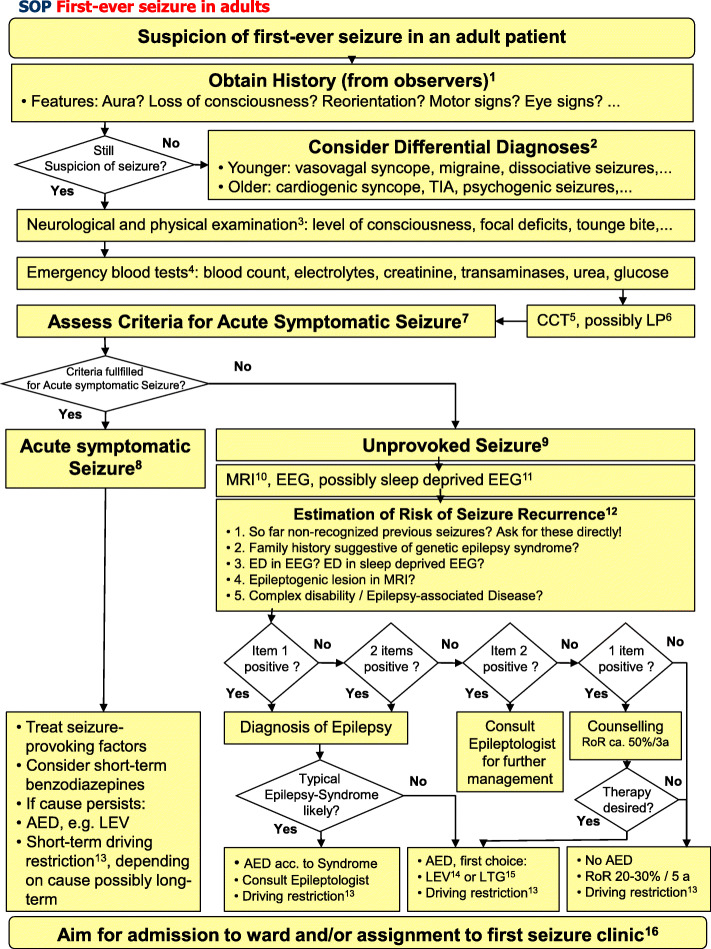
Suggestion of a step-wise standardized approach to a first-ever epileptipc seizure in adults. For abbreviations, see list at end of article

### Comments, explanations, additions (see footnotes in flow chart)

1. Quite often, the seizure has not been witnessed by the treating physician himself, and not even the emergency physician or paramedics presenting the patient. It is then of paramount importance to obtain a detailed description from eye witnesses. Today, pictures or movies taken by smartphones may also be very helpful. This may allow to recognize particular features of a seizure (i.e. involuntary repetitive and/or extending motoric and/or sensoric phenomena with or without disturbance or loss of consciousness) and even differentiate the type (generalized / focal, tonic / clonic / tonic-clonic /..., etc.). It should especially be asked whether the seizure was focally initiated (e.g. by head turn, which strongly suggests a focal onset) and how the eyes appeared (closed: rather non-epileptic seizure, upward deviation: rather syncope, open / “staring” and mid-position or sideward deviation: rather epileptic seizure). Another very important feature is whether the patient displayed a delayed or even prolonged re-awakening and re-orientation, which often reflects the post-ictal phase after a true epileptic seizure.
2. Misdiagnosis in suspected seizures is common (20–30%). Differential diagnoses may be grouped according to the age of the patient: In young adults, these may be vasovagal syncope, narcolepsy, paroxysmal dyskinesia, Tic, migraine with aura, panic attacks, hypnic myoclonus as a physiological sleep related phenomenon. In the elderlies, these may rather be cardiogenic syncope, transient ischemic attack, transient global amnesia, drop attacks, delirium, and toxic-metabolic encephalopathies, as well as REM- / Non-REM sleeping disorder. In both age groups, dissociative seizures (non-epileptic / psychogenic seizures) may occur (estimated incidence 5–30 / 100,000) and pose the greatest challenge in differentiation. Of note, the majority of syncopes is convulsive! See Table 1 for an overview of more frequent differential diagnoses (Table 1)

3. The neurological examination may yield ongoing signs of seizure activity (tonic / myoclonic convulsions, (orofacial) automatisms, loss of consciousness, etc.), postictal disturbance of consciousness, focal deficits indicative of an epileptogenic brain lesion, or be absolutely normal. A systemic physical inspection and examination may yield indicators of seizure-provoking factors (infection, drug / alcohol abuse, chronic disease, etc.), traumatic consequences of a seizure (fractures, luxations, bruises, open injuries, etc.), and possible seizure-assocations (enuresis, tounge bite (laterally!), muscle soreness, etc.), although the latter are often non-specific.
4. Emergency laboratory work-up may yield infection-related, metabolic or electrolyte derangements that may have triggered the seizure. These may constitute a criterion for an acute symptomatic seizure [7]. Drug abuse or toxicity may cause about 3% of first-ever seizures and may call for toxicology screening if suspected. In the past, prolactine levels were sometimes measured as an indicator for a true epileptic seizure, however, these turned out to be non-specific. Creatinine kinase may rise to indicate a convulsive seizure, but this can occur quite delayed and is, again, non-specific, as it may just be associated with trauma from a fall or convulsions from a syncope. It should still be part of the blood test to be integrated with other results. In the case of a series of seizures or even status epilepticus, creatinine kinase may rise massively and reach dangerous levels demanding nephroprotective fluid therapy.
5. Every patient with a first-ever seizure should receive cerebral imaging. If magnetic resonsance imaging (MRI) is not available acutely, as is often the case, cranial computed tomography (CCT) should be applied to rule out or detect direct or indirect signs of pathologies such as acute ischemic or hemorrhagic stroke, large tumor, CVST, etc. Its average yield of cerebral abnormalities after a first seizure is about 10%. CCT in the emergency room should definitely be done in patients older than 40 years, partial onset seizures, ongoing altered mental state, recent trauma, known cancer, anticoagulation, immunodeficiency, fever, and focal neurological deficits. MRI should then be added as soon as feasible.
6. Clinical and/or laboratory signs of meningitis or encephalitis demand a lumbar puncture. Initial basic CSF work-up for infections may later be followed by antineuronal antibody-tests in suspected autoimmune-encephalitis, hence portions of CSF should be stored.
7. Acute symptomatic seizures (ASS) have an incidence of about 20–30 / 100,000 and constitute about 40% of all afebrile seizures in the population. Suggested criteria for an ASS are: 1. Acute ischemic stroke / intracerebral hemorrhage; 2. Traumatic brain injury; 3. Hypoxic encephalopathy; 4. Post intracranial surgery; 5. Subdural hematoma, 6. Acute CNS infection or inflammation; 7. Metabolic derangement; 8. Drug / alcohol withdrawal (last consumption 7-48 h ago) [7]. Of note, sleep deprivation, although a well-known seizure-provoking factor, does not belong to these criteria. Because it was shown that the recurrence rate of seizures following a first seizure with such trigger is considerably higher than that of ASS, the International League Against Epilepsy (ILAE) recommends against considering seizures following sleep-deprivation as “provoked” seizures.
8. ASS have a similar life-time prevalence as unprovoked seizures (10%). In 20–30% of cases, ASS may constitute the beginning of an epilepsy, and patients with a known epilepsy syndrome may present with additional ASS. Antiepileptic drug (AED) therapy is not always necessary in ASS if the causative factor can be removed. If not, or at least temporarily, AED may be initiated and those with few side effects and interactions are to be preferred.
9. Whether or not AED treatment should be started after a first unprovoked seizure is a current controversial debate. That decision is always an individual one and carries a lot responsibility. This remains true despite more recent and more directive recommendations have been published, e.g. to treat seizures associated with a lesion on MRI or clear epileptiform discharges on EEG (5). The advantages to prevent further seizures have to be balanced against potential side effects of a life-long therapy that may not be necessary. Although AED treatment clearly reduces seizure recurrence within the next two years – which may be very relevant to an individual patient – it does not seem to change the long-term course of epilepsy [5].
10. Soon after the first seizure, magnetic resonance imaging (MRI) should be performed, since its yield of seizure-causing lesions (e.g. mesial temporal sclerosis, cortical dysplasias, arteriovenous malformations, microangiopathies, or tumors) is clearly higher than that of CCT. For a suggested MRI protocol, see ASA Table 2 (Table 2, see also [9]).
11. Electroencephalography (EEG) may start with a standard rountine investigation. The yield of abnormalities in a routine EEG is about 50% within the first 24 h and about 30% beyond that time window. Only clear epileptiform discharges (ED) that can be differentiated from artifacts in association with clinical seizures have a diagnostic and prognostic meaning. The absence of ED does not exclude the presence of epilepsy. The sensitivity of four standard EEGs in seizure-free intervals is about 70–80%. Instead of adding more standard EEGs after normal findings, a sleep deprived EEG or a long-duration video-EEG should rather be applied to enhance the yield. A sleep deprived EEG may raise a 40% yield of a routine EEG to about 60%.
12. The flow chart contains a simplified suggestion for estimation of the risk of recurrence. A more recent meta-analysis of ten high-quality studies found an overall risk of recurrence after a first unprovoked seizure of 37% at 2 years. The following four factors were particularly predictive (relative risk): prior brain insult (2.6), epileptiform abnormailties on EEG (2.2), abnormal brain imaging (2.1), and nocturnal seizures (2.1, however 95% CI 1.0–4.3). These results were incorporated in a recent guideline on that matter [10]. On direct questioning, about a third of patients with an assumedly first seizure have had absences, myoclonus, or auras in the past. Obtaining a detailed family history and a peripartal history is crucial, too, although this should certainly not be the only factor to drive AED treatment of a first seizure.
13. Driving restrictions after a first seizure differ considerably from country to country and even between states in a particular country. A reasonable driving restriction after an ASS may be 3–6 months while it may be one or more year(s) after an unprovoked seizure depending on the estimated recurrence risk. However, local regulations have to be followed. The physician is obliged to address that issue and recommended to keep written documentation of his recommendations. Counselling about safety issues regarding the behaviour in certain environments is also a very important part of the work-up and includes advice against working heights above 1 m, unattended climbing, swimming and taking a bath, using front hotplates when cooking, slippery floor covers in the bath-room, etc. Counselling should also include the phenomenon of sudden unexpected death in epilepsy (SUDEP) [11], although that may be debatable at a low risk of recurrence.
14. Levetiracetam: Virtually no relevant interactions. Side effects: Increased irritability and other psychological disturbances; demands lower dosing in renal insufficiency.
15. Lamotrigine: Requires low starting dose and slow increase. Side effects: skin rash, most extremely in Stevens-Johnson-syndrome, interaction with paracetamol and estrogen-containing contraceptives; demands tight level-control in pregnancy.
16. If the patient cannot be admitted to hospital for diagnostics to estimate the recurrence risk after an unprovoked seizure, he may be discharged after laboratory tests and CCT with the recommendation to timely obtain an outpatient MRI, EEG and neurology consultation and should optimally re-present to a first seizure clinic.

**Table 1:** Selected differential diagnoses (modified from [12])

	Epileptic Seizure	Syncope	Dissociative Seizure	Parasomnia	Paroxysmal Movement Disorder	Migraine (aura)
Duration	30–120 s	10–30 s	seconds to hours	seconds to minutes	seconds to hours	4 to 72 h
Eyes	open	open	(tightly) closed	open	open	open
Motor signs	automatisms, tonic, clonic, versive, tonic-clonic	irregular myoclonic and tonic convulsions	crescendo and decrescendo, variability from event to event	few targeted automatisms	dystonia, dyskinesia (athetotic, choreatic)	normal (exception: hemiplegic migraine)
Speech	ictal aphasia (dominant hemisphere)	no abnormalities after syncope	stuttering	no abnormality	possibly abnormality	no abnormality
Initiation	possibly aura (seconds)	vegetative prodromi (min.)	varies, possibly prodromal spells	none	tension oder paraesthesia	possibly aura
Re-orientation	often delayed	rapidly	often delayed and “stuttering”	none if returns to sleep	rapidly	gradually
EEG	epileptiform discharges (or normal)	normal (or general slowing)	normal	certain patterns from Non-REM or REM-sleep	normal	normal (or non-specific slowing)
Triggers	rarely, then stereotype (e.g., flicker light)	shock, pain, micturition, etc.	suggestion	drugs	possibly physical activity, coffeine, tea, training	stress, hormones, red wine, etc.

**Table 2:** Suggestions for an MRI protocol after first seizure or in epilepsy. Slice width 4 mm and less, contrast medium if lesion is found

Sequence	Slice	Orientation
T1	sagittal	standard
T2-TSE	axial	standard
FLAIR	axial/coronal	standard
T1	coronal	standard
T2-TSE	coronal	temporally angulated

## Conclusion

The approach to a suspected first epileptic seizure in adults should start with obtaining observer information to judge that suspicion. In a second step, diagnostic work-up of the seizure should include neurologic examination, emergency blood tests, and cerebral imaging to then differentiate an acute symptomatic seizure from an unprovoked seizure. In the former, identification and -if possible- treatment of the acute underlying cause should dominate the management. In the latter, the focus should be on estimation of risk of seizure recurrence and the decision to start antiepileptic treatment.

## Abbreviations

AED: Antiepileptic drug(s)
ASS: Acute symptomatic seizure
CCT: Cranial Computed Tomography
CVST: Cerebral venous and sinus thrombosis
ED: Epileptiform discharges
EEG: Electroencephalography / electroencephalogram
HE: Hypoxic encephalopathy
ILAE: International League against Epilepsy
LEV: Levetiracetam
LP: Lumbar puncture
LTG: Lamotrigine
MRI: Magnetic resonance imaging
REM: Rapid Eye Movement
RoR: Risk of Recurrence
SOP: Standard operating procedure
SUDEP: Sudden unexpected death in epilepsy
